# Stroke seven hours after SARS-CoV-2 vaccination

**DOI:** 10.1016/j.clinsp.2023.100193

**Published:** 2023-04-10

**Authors:** Ana Claudia Fiorini, Carla Alexandra Scorza, Antonio-Carlos G. de Almeida, Fulvio Alexandre Scorza, Josef Finsterer

**Affiliations:** a Programa de Estudos Pós-Graduado em Fonoaudiologia, Pontifícia Universidade Católica de São Paulo (PUC-SP), São Paulo, SP, Brazil; b Departamento de Fonoaudiologia, Escola Paulista de Medicina, Universidade Federal de São Paulo (EPM/UNIFESP), São Paulo, SP, Brazil; c Disciplina de Neurociência, Universidade Federal de São Paulo/Escola Paulista de Medicina (UNIFESP/EPM), São Paulo, SP, Brazil; d Centro de Neurociências e Saúde da Mulher “Professor Geraldo Rodrigues de Lima”, Escola Paulista de Medicina/Universidade Federal de São Paulo (EPM/UNIFESP), São Paulo, SP, Brazil; e Neurology & Neurophysiology Center, Vienna, Austria

Since the introduction of SARS-CoV-2 vaccines, adverse reactions have occasionally been reported for all types of SARS-CoV-2 vaccines 1, 2, 3. One of these rare side effects is ischemic stroke, which usually occurs a few days after SARS-CoV-2 vaccinations [3]. Ischemic stroke a few hours after vaccination with an mRNA-based SARS-CoV-2 vaccine has been reported only rarely.

The patient is a 73-year-old, non-smoking, HIV-negative female, height 167 cm, weight 57 kg, who developed acute-onset of right lower limb weakness 7 hours after the first vaccination with an mRNA-based SARS-CoV-2 vaccine. She was admitted two days later. Her history was positive for prior smoking, prior arterial hypertension, hyperlipidemia, right-sided nodular goiter, ovariectomy, hysterectomy at age 43, resection of a basal cell carcinoma at age 70, lumbar disc prolapses not requiring surgery, and right unilateral total hip replacement at age 73. Until admission, she received medication with acetyl-salicylic acid, valsartan, amlodipine, and calcium. Blood pressure and serum lipids were constantly within normal range before admission. Her mother's history was positive for aortic dissection, epilepsy, and short stature, but non-informative to other family members.

On clinical neurologic examination, the patient presented with mild right upper limb weakness (M5-), right lower limb weakness (M5-), right lower limb ataxia, a right extensor plantar response, and pronounced tendon reflexes of the right lower limb. The blood pressure was 120/90 mmHg. The ECG showed sinus rhythm and only left ventricular hypertrophy. Blood tests revealed mild hypercholesterolemia, lambda-type monoclonal paraprotein, and HbA1c of 6.0% (n, 4%‒5.7%). The D-dimer was 0.61 mg/L (n, < 0.5 mg/L). Cerebral Magnetic Resonance Imaging (MRI) showed a partial, subacute ischemic lesion in the left middle cerebral artery territory (Fig. 1). MR-angiography revealed an aneurysm (10 mm) of the anterior communicating artery, but no stenosis or occlusion of an intracerebral artery (Fig. 1). Carotid ultrasound showed only mild atherosclerosis. The antithrombotic therapy was switched to clopidogrel. Under this therapy and physiotherapy, the hemiparesis completely disappeared within a few days and the patient was transferred to a rehabilitation unit with a modified Rankin Scale (mRS) of 0.

**Fig. 1:**
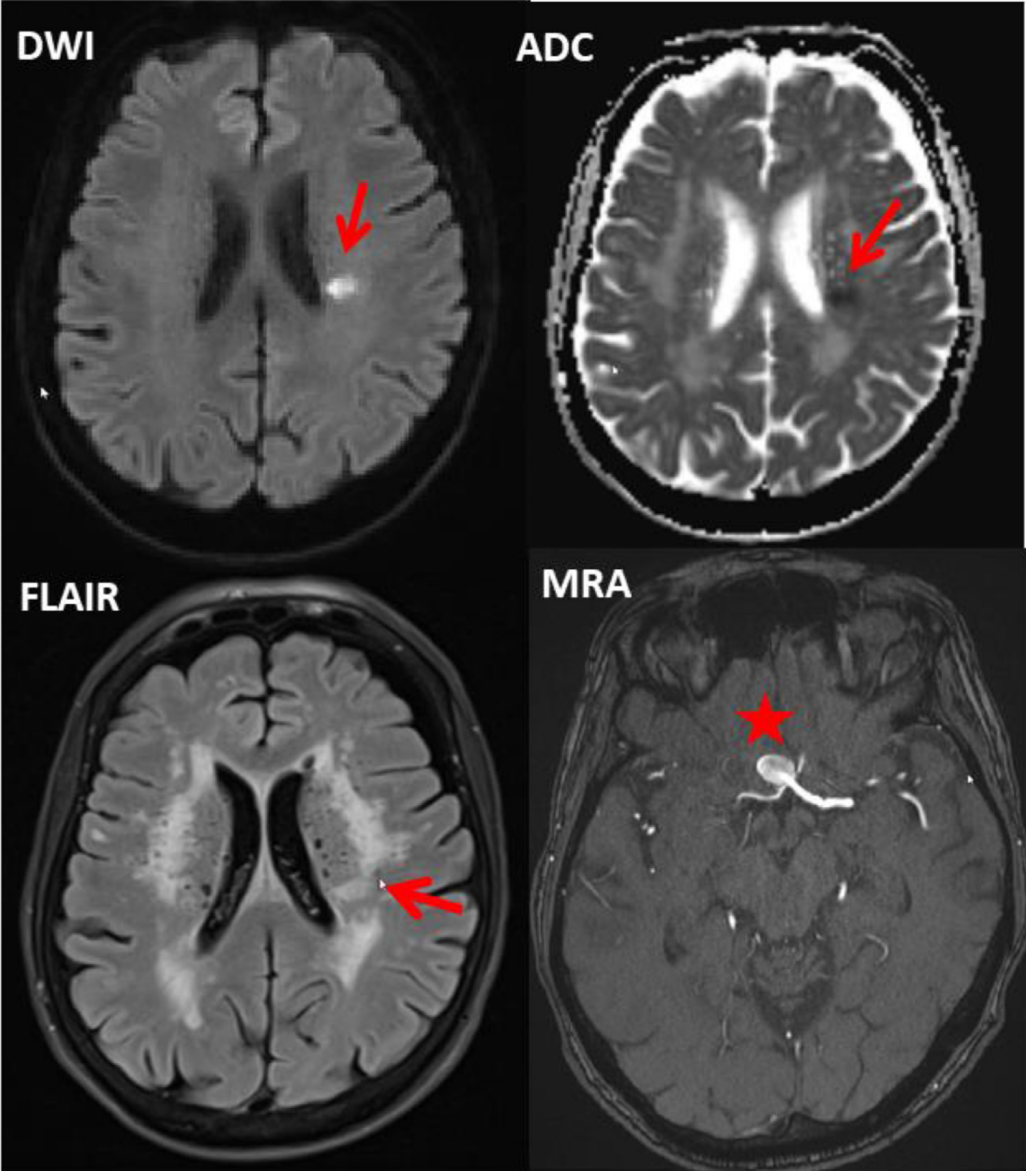
Cerebral MRI of the index patient showing an acute ischemic lesion periventricularly on the left side (arrows), leucoaraiosis, and an aneurysm of the communicant anterior artery (asterik) with 10 mm in diameter (DWI, Diffusion Weighted Imaging; ADC, Apparent Diffusion Coefficient; FLAIR, Fluid Attenuated Inversion Recovery; MRA, MR-Angiography).

The case is notable for an ischemic stroke seven hours after the first dose of an mRNA-based SARS-CoV-2 vaccine. Whether there was a causal relationship between the vaccination and the stroke remains speculative but is conceivable since the patient's cardiovascular risk factors were well controlled. Arguments for a causal relationship are that the stroke occurred time-linked to the vaccination, that ischemic and hemorrhagic strokes have already been reported as complications of SARS-CoV-2 vaccinations 4, 5, 6, 7, that stroke even shortly after SARS-CoV-2 vaccinations has been reported [4], that thrombus formation in both the arterial and venous circulation is increasingly being recognized as a complication of SARS-CoV-2 vaccinations [8], and that ischemic stroke also occurred after varicella and influenza vaccinations [4]. Arguments against a causal relationship are that the patient had classical cardiovascular risk factors, such as arterial hypertension, and hyperlipidemia, but both were well-controlled prior to admission and throughout the hospital stay

Although stroke has occasionally been reported as a complication of SARS-CoV-2 vaccinations 3, 4, 5, 6, 7, the pathophysiological mechanism underlying stroke occurrence is poorly understood. Putative pathophysiologic mechanisms of a vaccination-associated stroke include hypoperfusion due to endothelialitis, cerebral vasculitis, Reversible, Cerebral Vasoconstriction Syndrome (RCVS), or dissection, and thrombus formation due to endocarditis, myocarditis, atrial fibrillation, heart failure, or hypercoagulability (Vaccination Induced Thrombotic Thrombocytopenia [VITT]) in response to the reaction of the immune system against the virus (cytokinetic storm) [4]. Although cardiac involvement is a well-known complication of SARS-CoV-2 infection, it has been reported less frequently after SARS-CoV-2 vaccinations. Common side effects of SARS-CoV-2 vaccines reported so far include allergic reactions [8], lymphadenopathy [1], deep vein thrombosis[10] dizziness, headache, pain, muscle spasms, myalgia, paresthesia [3], Venous Sinus Thrombosis (VST) [9], Guillain-Barre Syndrome (GBS), Small Fiber Neuropathy (SFN), and immune encephalitis [10]. Rarely, tremors, diplopia, tinnitus, dysphonia, transverse myelitis, facial palsy, seizures, stroke, and Acute Disseminated Encephalomyelitis (ADEM) have been reported [3]. An immunological reaction to the vaccine is unlikely to explain the occurrence of vaccine-associated stroke, as such a response is not expected within a few hours after vaccination.

In summary, an acute, ischemic stroke can occur shortly after a SARS-CoV-2 vaccination. There are more arguments for than against a causal relationship between vaccination and stroke. Future anti-SARS-CoV-2 vaccines must become safer for everyone who is vaccinated.

## Declarations

Funding sources: No funding was received.

Acknowledgments: None.

Ethics approval: Was in accordance with ethical guidelines. The study was approved by the institutional review board.

Consent to participate: Was obtained from the patient.

Consent for publication: Informed consent was obtained from the patient.

Availability of data: All data are available from the corresponding author.

Code availability: Not applicable.

## Authors' contributions

ACF, CAS, AGA, and FAS: literature search, discussion, final approval; JF: Design, literature search, discussion, first draft, critical comments, final approval.

## Conflicts of interest

The authors declare no conflicts of interest.
